# Federated Logistic Regression with Enhanced Privacy: A Dynamic Gaussian Perturbation Approach via ADMM from an Information-Theoretic Perspective

**DOI:** 10.3390/e27111148

**Published:** 2025-11-12

**Authors:** Jie Yuan, Yue Wang, Hao Ma, Wentao Liu

**Affiliations:** 1 School of Automation, Wuxi University, Wuxi 214122, China; 2 Department of Science and Technology, Wuxi University, Wuxi 214122, China; 3 School of Internet of Things Engineering, Jiangnan University, Wuxi 214122, China; 4 College of Automation and Electronic Engineering, Qingdao University of Science and Technology, Qingdao 266061, China

**Keywords:** federated learning, differential privacy, logistic regression, Gaussian mechanism, ADMM

## Abstract

Federated learning enables distributed model training across edge nodes without direct raw data sharing, but model parameter transmission still poses significant privacy risks. To address this vulnerability, a Distributed Logistic Regression Gaussian Perturbation (DLGP) algorithm is proposed, which integrates the Alternating Direction Method of Multipliers (ADMM) with a calibrated differential privacy mechanism. The centralized logistic regression problem is decomposed into local subproblems that are solved independently on edge nodes, where only perturbed model parameters are shared with a central server. The Gaussian noise injection mechanism is designed to optimize the privacy–utility trade-off by introducing calibrated uncertainty into parameter updates, effectively obscuring sensitive information while preserving essential model characteristics. The $\ell_{2}$-sensitivity of local updates is derived, and a rigorous (ϵ,δ)-differential privacy guarantee is provided. Evaluations are conducted on a real-world dataset, and it is demonstrated that DLGP maintains favorable performance across varying privacy budgets, numbers of nodes, and penalty parameters.

## 1. Introduction

Federated learning (FL) has emerged as a transformative paradigm for distributed machine learning, enabling collaborative model training across edge nodes while preserving data privacy by avoiding raw data transmission [1,2]. However, the exchange of model parameters between edge nodes and a central server introduces new privacy risks. Recent studies have demonstrated that adversaries can reverse-engineer sensitive information from model updates, such as training data features and label distributions [3]. This vulnerability arises because gradient updates and parameter vectors inherently encode statistical properties of the underlying datasets [4]. For example, the work of [5] showed that adversarial models can reconstruct high-fidelity approximations of training images from shared model updates, highlighting the inadequacy of FL’s native privacy guarantees. From an information-theoretic perspective, this vulnerability stems from the excessive information content, or low entropy, of the model updates, which can be exploited by adversaries to infer sensitive details about the training data. Additionally, the centralized aggregation architecture often exacerbates communication bottlenecks and computational burdens, particularly in bandwidth-constrained edge environments [6,7]. These challenges necessitate the development of robust privacy-preserving mechanisms that can mitigate information leakage during parameter transmission while maintaining computational efficiency.

To address privacy risks in FL, differential privacy (DP) has been widely adopted as a rigorous framework for quantifying and bounding information leakage [8,9]. Existing approaches typically inject calibrated noise into model parameters or gradients using mechanisms like the Gaussian mechanism [10,11] or Laplace mechanism [12,13], ensuring that individual data contributions remain indistinguishable. For instance, ref. [14] proposed a DP-regularized FL framework where noise is added to client updates before aggregation, achieving (ϵ,δ)-differential privacy. The Gaussian mechanism, in particular, operates by increasing the entropy of the output distribution, making it statistically difficult to distinguish between outputs from adjacent datasets. Concurrently, distributed optimization techniques like the Alternating Direction Method of Multipliers (ADMM) have been leveraged to decompose global optimization tasks into parallelizable local subproblems, reducing reliance on centralized computation [15,16]. ADMM-based FL frameworks enable edge nodes to optimize local models independently while maintaining consistency through iterative parameter exchanges [17,18]. However, integrating DP with ADMM remains non-trivial, as the dynamic nature of distributed updates complicates sensitivity analysis and noise calibration [19,20]. Furthermore, many existing methods focus on static environments and fail to account for dynamic client dropout or heterogeneous data distributions, leading to degraded privacy–utility trade-offs [21,22].

Despite significant progress, several critical challenges persist in current FL privacy-preserving frameworks. First, most DP mechanisms assume homogeneous data distributions and static client participation, which rarely hold in real-world edge scenarios. For example, client dropout during training can invalidate privacy budgets and introduce statistical biases, yet existing solutions often lack resilience to such dynamics [23]. Second, the computational overhead of DP mechanisms, particularly for high-dimensional models, remains prohibitive for resource-constrained edge devices. Existing works rely on Laplace noise, which incurs higher variance than Gaussian noise for equivalent privacy guarantees, degrading model accuracy [24]. Third, the sensitivity analysis required for noise calibration is often oversimplified, leading to either insufficient privacy protection or excessive noise that undermines model utility. This directly impacts the information-theoretic properties of the mechanism, as miscalibrated noise fails to provide sufficient entropy to mask individual contributions effectively. Finally, current frameworks often neglect the communication efficiency challenges inherent in edge environments, where frequent parameter exchanges can strain bandwidth and latency constraints.

This paper presents the Distributed Logistic Regression Gaussian Perturbation (DLGP) algorithm, a novel FL framework that addresses these gaps by seamlessly integrating ADMM with differential privacy. The key contributions are as follows:

- DLGP employs ADMM to decompose centralized logistic regression into parallel local optimizations, allowing edge nodes to retain raw data while sharing only aggregated parameters. This architecture minimizes communication overhead and computational burdens on the central server.
- Through rigorous $\ell_{2}$-sensitivity analysis of local parameter updates, DLGP dynamically calibrates Gaussian noise to achieve (ϵ,δ)-differential privacy. Unlike static noise injection methods, DLGP adjusts noise levels based on local dataset sizes and model sensitivity, ensuring optimal privacy–utility balance across heterogeneous edge nodes.
- By perturbing parameters at edge nodes before transmission, DLGP eliminates the need for a trusted central server and mitigates risks of server-side data leakage. This design aligns with the decentralized nature of edge computing and enhances resilience to adversarial attacks.
- Experimental evaluations on a real-world occupancy detection dataset demonstrate that DLGP maintains favorable empirical loss performance across varying privacy budgets and edge node configurations, validating its practical effectiveness in balancing privacy preservation and model utility.

The remainder of this paper is structured as follows: Section 2 introduces theoretical foundations including distributed logistic regression, differential privacy, and ADMM. Section 3 describes the ADMM-based distributed training framework. Section 4 presents the DLGP algorithm with its adaptive noise injection mechanism. Section 5 provides formal privacy guarantees. Section 6 reports experimental results. Finally, Section 7 reiterates the core contributions of the DLGP algorithm and synthesizes its implications for privacy-preserving federated learning.

## 2. Model Description and Preliminaries

This section establishes the theoretical foundation for the proposed privacy-preserving federated learning framework. The formulation of logistic regression models in both centralized and distributed paradigms is first elaborated, followed by an introduction to the core concepts of differential privacy and the ADMM, which form the basis of the proposed algorithm design. These foundational concepts are examined through an information-theoretic lens, particularly focusing on how they manage and protect information content throughout the learning process.

### 2.1. Distributed Logistic Regression Model

Logistic regression remains a cornerstone of statistical learning for binary classification tasks, with the goal of learning a parameter vector that maps input features to binary labels. In federated learning scenarios, the transition from centralized to distributed modeling is pivotal for mitigating raw data exposure risks, necessitating a clear distinction between these two paradigms upfront.

In the centralized setting, all training data is aggregated at a central server, where the model is trained by minimizing a regularized empirical risk function. For a global dataset $D=\{\left(x_{ij},y_{ij}\right),i=1,2,\cdots ,n;j=1,2,\cdots ,m_{i}\}$, the centralized logistic regression objective function minimizes the regularized empirical risk as

(1) $$\min_{\alpha }\sum_{i=1}^{n}\sum_{j=1}^{m_{i}}\frac{1}{m_{i}}\log\left(1+\exp\left(-y_{ij}\alpha^{T}x_{ij}\right)\right)+\frac{\lambda }{2}{∥\alpha ∥}_{2}^{2},$$

where $\alpha \in R^{d}$ denotes the global model parameter, $x_{ij}\in R^{d}$ represents the *d*-dimensional feature vectors, and $y_{ij}\in \left\{-1,1\right\}$ is the binary label with −1 and 1 indicating distinct classes such as machine normal/faulty states or user absence/presence. The term λ is a regularization parameter, and ${∥\alpha ∥}_{2}$ denotes the $\ell_{2}$ norm to prevent overfitting. However, this centralized approach concentrates the information content (and thus the risk) of the entire dataset into a single model, resulting in a low-entropy system that is highly vulnerable to privacy attacks. The key notations used throughout this chapter are summarized in Table 1. Despite its simplicity, this paradigm exhibits critical limitations in privacy-sensitive environments. Raw data must be uploaded to a central server, which creates vulnerabilities to sensitive information breaches. Additionally, aggregating large-scale data from edge nodes introduces prohibitive communication overhead and latency, making it particularly challenging for deployment in bandwidth-constrained edge computing environments [25].

**Figure 1:**
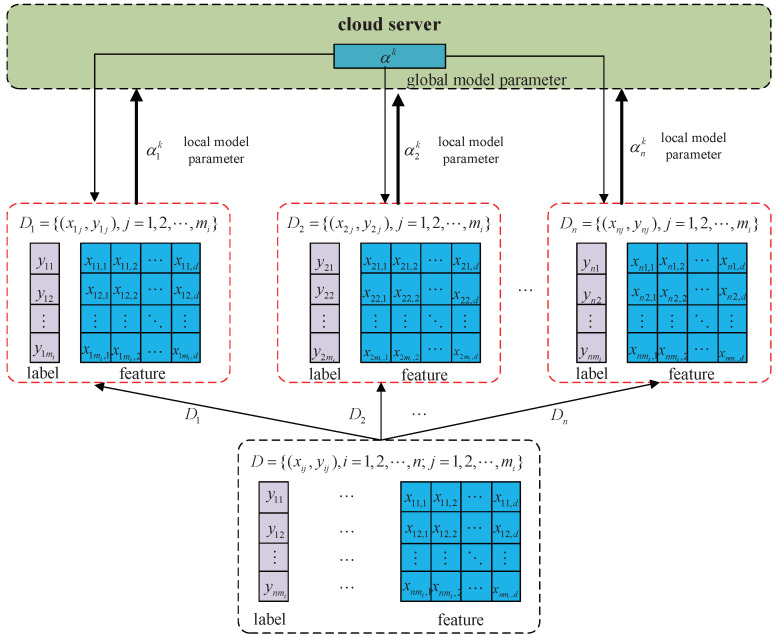
Privacy-preserving distributed logistic regression model.

To address these limitations, distributed logistic regression decomposes the global learning task across edge nodes, where each node retains its local data and shares only model parameters with the central server. Consider a federated system consisting of *n* edge nodes, where node *i* maintains a local dataset $D_{i}={\left\{\left(x_{ij},y_{ij}\right)\right\}}_{j=1}^{m_{i}}$ containing $m_{i}$ samples. Through the decentralization of the centralized strategy, Equation (1) can be reformulated as

(2) $$\min_{{\left\{\alpha_{i}\right\}}_{i=1}^{n}}\sum_{i=1}^{n}\left(\sum_{j=1}^{m_{i}}\frac{1}{m_{i}}\log\left(1+\exp\left(-y_{ij}\alpha_{i}^{T}x_{ij}\right)\right)+\frac{\lambda }{2n}{∥\alpha_{i}∥}_{2}^{2}\right),\;s.t.\;\alpha_{i}=\alpha ,$$

where ${\left\{\alpha_{i}\right\}}_{i=1}^{n}=\left\{\alpha_{1},\alpha_{2},\cdots ,\alpha_{n}\right\}$ denotes the set of local parameters, with $\alpha_{i}\in R^{d}$ representing the local model parameter at edge node *i* and $\alpha \in R^{d}$ denoting the global model parameter optimized across all nodes. This distributed approach inherently increases the system’s entropy by dispersing information across multiple edge nodes, thereby reducing the risk of catastrophic privacy breaches that could occur in a centralized setting. As illustrated in Figure 1, this centralized learning task is split across *n* edge nodes for parallel processing. In the *k*-th iteration, each edge node uploads its local model parameter $\alpha_{i}^{k}$ to the cloud server, where the collected local parameters are aggregated to form the global model parameter $\alpha^{k}$ before being made available for download. Each edge node then leverages the downloaded global parameter to resume local model training and uploads its (k+1)-th local model parameter to the cloud server. This iterative process continues, enabling alternating updates between local and global parameters. Eventually, all local model parameters across the edge nodes converge to the global model parameter α, satisfying the constraint outlined in Equation (2).

### 2.2. Differential Privacy

To protect model parameters during communication against privacy inference attacks, differential privacy is employed as a rigorous framework. It quantifies the indistinguishability of algorithm outputs with respect to individual data samples, ensuring robust privacy guarantees. This indistinguishability criterion directly relates to information-theoretic concepts of uncertainty and entropy, as it ensures that the output distribution maintains sufficient randomness to prevent confident inferences about any individual data point.

**Definition** **1.** *(ϵ,δ)-Differential Privacy [9]: Let D and $D^{\prime }$ be two adjacent datasets that differ in at most one record. For a randomized algorithm A, let Range(A) denote the set of all possible outputs of the algorithm on these adjacent datasets. The algorithm A satisfies (ϵ,δ)-differential privacy if, for any output subset O⊆Range(A), the condition*

(3) $$\mathrm{Pr}\left[A\left(D\right)\in O\right]\leq \exp\left(\epsilon \right)\cdot \mathrm{Pr}\left[A\left(D^{\prime }\right)\in O\right]+\delta$$

*holds.*

The selection of parameters ϵ and δ depends on specific privacy policies. The parameter ϵ represents the privacy budget, which reflects the degree of privacy protection. The relaxation probability δ controls the probability of privacy loss. When δ=0, the algorithm A satisfies ϵ-differential privacy [8]. The most commonly used privacy-preserving mechanism to achieve (ϵ,δ)-differential privacy is the Gaussian mechanism, which employs data perturbation techniques by adding Gaussian noise to query results. The perturbation effectively increases the entropy of the output, making it more difficult for adversaries to extract useful information about individual data points while preserving the overall statistical utility of the model.

**Definition** **2.** *$\ell_{2}$-Sensitivity [9]: Let D and $D^{\prime }$ be two adjacent datasets that differ in at most one record. For a query function q, its $\ell_{2}$-sensitivity is defined as*

(4) $$s=\max_{D,D^{\prime }}{∥q\left(D\right)-q\left(D^{\prime }\right)∥}_{2},$$

*where ${∥\cdot ∥}_{2}$ denotes the $\ell_{2}$ norm.*

**Definition** **3.** *Gaussian Mechanism [9]: Given a dataset D and a privacy budget ϵ∈(0,1), a randomized algorithm A provides (ϵ,δ)-differential privacy for a query function q if and only if*

(5) A(D)=q(D)+Gau(σ),

*where σ≥cs/ϵ with $c^{2}>2\ln\left(1.25/\delta \right)$, Gau(σ) denotes Gaussian noise with zero mean and variance $\sigma^{2}$, and s represents the $\ell_{2}$-sensitivity.*

The Gaussian mechanism is well-suited for federated learning, efficiently perturbing high-dimensional parameters with manageable computational overhead. From an information-theoretic perspective, the Gaussian noise maximizes entropy for a given variance, providing the most uncertainty about the original data for a given noise power constraint, which aligns optimally with privacy preservation goals.

### 2.3. Alternating Direction Method of Multipliers

To solve constrained optimization problems in distributed systems, the ADMM offers a powerful framework by decomposing complex objectives into tractable subproblems [26,27]. This decomposition is particularly advantageous for federated learning scenarios, where data remains decentralized across edge nodes. The general form of optimization problems addressed by ADMM is

(6) minf(p)+g(q)s.t.Ap+Bq=C,

where *f* and *g* are convex objective functions, *p* and *q* denote primal variables, and *A*, *B*, and *C* are parameters defining the equality constraint. The augmented Lagrangian function for the problem in (6) is

(7) $$L\left(p,q,\gamma \right)=f\left(p\right)+g\left(q\right)+\langle \gamma ,Ap+Bq-C\rangle +\frac{\mu }{2}{∥Ap+Bq-C∥}_{2}^{2},$$

where γ≥0 represents the dual variable and μ>0 is a penalty parameter that controls the strength of constraint enforcement.

ADMM solves the optimization problem through an iterative process involving three alternating updates. In each iteration, the primal and dual variables are updated sequentially as

(8) $$\begin{array}{ccc} p^{k+1} & = & \arg\min_{p}L\left(p,q^{k},\gamma^{k}\right), \end{array}$$

(9) $$\begin{array}{ccc} q^{k+1} & = & \arg\min_{q}L\left(p^{k+1},q,\gamma^{k}\right), \end{array}$$

(10) $$\begin{array}{ccc} \gamma^{k+1} & = & \gamma^{k}+\mu \left(Ap^{k+1}+Bq^{k+1}-C\right). \end{array}$$

As evident from the update rules in (8)–(10), the primal variables *p* and *q* and the dual variable γ are updated alternately in each iteration. This process continues until the algorithm meets predefined convergence criteria, ensuring that the solution satisfies both the objective function minimization and the equality constraint.

For our distributed logistic regression task described in Section 2.1, ADMM can be adapted to solve the constrained optimization problem in (2). By aligning the problem structure with the general ADMM framework, we decompose the global objective into local subproblems that can be solved in parallel across edge nodes, while maintaining consistency through the alternating update mechanism. This adaptation enables efficient decentralized computation, making it well-suited for privacy-preserving federated learning systems.

## 3. ADMM-Based Distributed Training Framework

Building upon the theoretical foundations of distributed optimization and differential privacy established in Section 2, this section presents the detailed implementation of our federated logistic regression framework. Leveraging ADMM, efficient distributed model training across edge nodes is enabled while strict consistency between local and global models is maintained. The distributed nature of this framework inherently promotes information dispersion, increasing the overall entropy of the learning system and making it more resilient to privacy attacks that exploit concentrated information patterns. The core workflow decomposes the global optimization task into parallel local computations, coordinated via a central server through iterative parameter exchanges, thereby eliminating the need for raw data transmission.

### 3.1. Distributed Model Formulation and Augmented Lagrangian Construction

The federated learning system under consideration comprises a central server and *n* edge nodes, with each edge node *i* maintaining a private local dataset $D_{i}={\left\{\left(x_{ij},y_{ij}\right)\right\}}_{j=1}^{m_{i}}$ containing $m_{i}$ samples. The global objective involves learning a consistent logistic regression model parameter $\alpha \in R^{d}$ that minimizes the aggregated empirical risk across all local datasets, without direct data sharing between nodes or with the server.

As formulated in Section 2, the distributed optimization problem given by Equation (2) enforces consistency between local parameters ${\left\{\alpha_{i}\right\}}_{i=1}^{n}$ and the global parameter α through equality constraints. To solve this constrained optimization problem using ADMM, the augmented Lagrangian function is constructed by extending the general form in Equation (7) as

(11) $$L\left({\left\{\alpha_{i}\right\}}_{i=1}^{n},\alpha ,{\left\{\gamma_{i}\right\}}_{i=1}^{n}\right)=\sum_{i=1}^{n}L_{i}\left(\alpha_{i},\alpha ,\gamma_{i}\right),$$

where the local component $L_{i}\left(\alpha_{i},\alpha ,\gamma_{i}\right)$ for each edge node *i* is defined as

(12) $$\begin{array}{ccc} L_{i}\left(\alpha_{i},\alpha ,\gamma_{i}\right) & = & \sum_{j=1}^{m_{i}}\frac{1}{m_{i}}\log\left(1+\exp\left(-y_{ij}\alpha_{i}^{T}x_{ij}\right)\right)+\frac{\lambda }{2n}{∥\alpha_{i}∥}_{2}^{2}\\ & & -\langle \gamma_{i},\alpha_{i}-\alpha \rangle +\frac{\mu }{2}{∥\alpha_{i}-\alpha ∥}_{2}^{2}. \end{array}$$

In this formulation, $\gamma_{i}$ denotes the dual variable (Lagrange multiplier) for enforcing the consistency constraint at node *i*, and μ>0 is the penalty parameter controlling the strength of constraint enforcement. The augmented Lagrangian integrates local loss minimization with constraint penalties, enabling decomposition into parallelizable local subproblems while maintaining global model consistency.

### 3.2. Edge Node Local Parameter Update

The ADMM training process initiates with local parameter updates at each edge node. In the *k*-th iteration, each node *i* optimizes its local parameter $\alpha_{i}$ by minimizing the local augmented Lagrangian component $L_{i}$, with optimization performed using the global parameter $\alpha^{k-1}$ and dual variable $\gamma_{i}^{k-1}$ from the previous iteration. This local optimization subproblem is expressed as

(13) $$\alpha_{i}^{k}=\arg\min_{\alpha_{i}}L_{i}\left(\alpha_{i},\alpha^{k-1},\gamma_{i}^{k-1}\right).$$

To solve this convex optimization problem efficiently, Newton’s method is employed, which leverages second-order gradient information to achieve faster convergence than first-order methods. For simplicity, the local cost function is defined as $J\left(\alpha_{i}\right)=L_{i}\left(\alpha_{i},\alpha^{k-1},\gamma_{i}^{k-1}\right)$. In each inner iteration *t*, $J(\alpha_{i})$ is approximated through a second-order Taylor expansion around $\alpha_{i}^{\left(t\right)}$, given by

(14) $$\begin{array}{ccc} J(\alpha_{i}) & = & J\left(\alpha_{i}^{\left(t\right)}\right)+{\left(\alpha_{i}-\alpha_{i}^{\left(t\right)}\right)}^{T}\nabla J\left(\alpha_{i}^{\left(t\right)}\right)+\frac{1}{2}{\left(\alpha_{i}-\alpha_{i}^{\left(t\right)}\right)}^{T}H\left(\alpha_{i}^{\left(t\right)}\right)\left(\alpha_{i}-\alpha_{i}^{\left(t\right)}\right)\\ & & +o\left(∥\alpha_{i}-\alpha_{i}^{\left(t\right)}{∥}^{2}\right), \end{array}$$

where $\nabla J(\alpha_{i}^{\left(t\right)})$ denotes the gradient and $H(\alpha_{i}^{\left(t\right)})$ denotes the Hessian matrix of $J(\alpha_{i})$ evaluated at $\alpha_{i}^{\left(t\right)}$. Minimizing this quadratic approximation yields the Newton update rule expressed as

(15) $$\alpha_{i}^{\left(t+1\right)}=\alpha_{i}^{\left(t\right)}-a_{t}{\left[H(\alpha_{i}^{\left(t\right)})\right]}^{-1}\nabla J\left(\alpha_{i}^{\left(t\right)}\right),$$

where $a_{t}$ denotes a step size determined through line search to ensure convergence. Specifically, this step size is computed as $a_{t}=\arg\min_{a\geq 0}J\left(\alpha_{i}^{\left(t\right)}-a{\left[H(\alpha_{i}^{\left(t\right)})\right]}^{-1}\nabla J\left(\alpha_{i}^{\left(t\right)}\right)\right)$.

For logistic regression, closed-form expressions for the gradient and Hessian are derived using only local data. The gradient is computed as

(16) $$\begin{array}{ccc} \nabla J(\alpha_{i}^{\left(t\right)}) & = & \sum_{j=1}^{m_{i}}\frac{1}{m_{i}}\frac{-y_{ij}x_{ij}}{1+\exp\left(y_{ij}{\left(\alpha_{i}^{\left(t\right)}\right)}^{T}x_{ij}\right)}+\frac{\lambda }{n}\alpha_{i}^{\left(t\right)}-\gamma_{i}^{k-1}+\mu \left(\alpha_{i}^{\left(t\right)}-\alpha^{k-1}\right), \end{array}$$

and the Hessian matrix is given by

(17) $$\begin{array}{ccc} H(\alpha_{i}^{\left(t\right)}) & = & \sum_{j=1}^{m_{i}}\frac{1}{m_{i}}\frac{y_{ij}^{2}x_{ij}x_{ij}^{T}\exp\left(y_{ij}{\left(\alpha_{i}^{\left(t\right)}\right)}^{T}x_{ij}\right)}{{\left(1+\exp\left(y_{ij}{\left(\alpha_{i}^{\left(t\right)}\right)}^{T}x_{ij}\right)\right)}^{2}}+\left(\frac{\lambda }{n}+\mu \right)I_{d}, \end{array}$$

where $I_{d}$ denotes the d×d identity matrix. Each edge node performs these inner Newton iterations until a local convergence criterion is satisfied, such as completion of $t_{\max}$ inner iterations. Upon convergence, the optimized local parameter $\alpha_{i}^{k}=\alpha_{i}^{\left(t_{\max}\right)}$ is prepared for upload to the central server.

### 3.3. Cloud Server Global Parameter Aggregation

Upon completion of local updates, all edge nodes upload their optimized local parameters ${\left\{\alpha_{i}^{k}\right\}}_{i=1}^{n}$ to the central server. In contrast to traditional centralized training paradigms that require direct transmission of raw data, this parameter-only communication strategy minimizes bandwidth consumption and mitigates privacy risks associated with sensitive data exposure.

Upon receiving the local parameters from all edge nodes, the central server performs global model aggregation to update the global parameter $\alpha^{k}$. Derived from the ADMM framework, the global aggregation rule integrates dual variables to balance constraint enforcement and model consistency, and is given by

(18) $$\alpha^{k}=\frac{1}{n}\sum_{i=1}^{n}\alpha_{i}^{k}-\frac{1}{n}\sum_{i=1}^{n}\frac{\gamma_{i}^{k-1}}{\mu }.$$

This aggregation mechanism ensures that the global parameter incorporates collective knowledge from all edge nodes while accounting for previous constraint violations, thereby driving the system toward a consistent solution. Following aggregation, the updated global parameter $\alpha^{k}$ is broadcast back to all edge nodes to guide their next round of local optimization.

### 3.4. Dual Variable Update

To strengthen constraint enforcement and accelerate convergence, each edge node updates its dual variable $\gamma_{i}$ based on the discrepancy between its local parameter and the aggregated global parameter. This update step is critical for maintaining the consistency constraint $\alpha_{i}=\alpha$ across iterations. Following the ADMM update mechanism outlined in Equation (10), the dual variable update for node *i* is given by

(19) $$\gamma_{i}^{k}=\gamma_{i}^{k-1}-\mu \left(\alpha_{i}^{k}-\alpha^{k}\right).$$

This adjustment modifies the Lagrange multiplier to penalize deviations between $\alpha_{i}^{k}$ and $\alpha^{k}$, where larger discrepancies result in more significant adjustments to $\gamma_{i}^{k}$ that in turn exert stronger corrective pressure on subsequent local optimization steps. As iterations progress, local parameters incrementally align with the global model, driving the system toward a consistent solution.

### 3.5. Complete Training Procedure

The complete ADMM-based distributed training process proceeds through iterative cycles of local parameter optimization, global aggregation, and dual variable updating, continuing until a predefined stopping criterion is satisfied (e.g., upon completing *K* total iterations). The detailed workflow of the algorithm is summarized in Algorithm 1.
**Algorithm 1** ADMM-Based Distributed Logistic Regression Training Algorithm**Require:** Local datasets $D={\left\{x_{ij},y_{ij}\right\}}_{i=1,\ldots ,n;j=1,\ldots ,m_{i}}$.**Ensure:** Final global model parameter $\alpha^{K}$.1:Initialize the global parameter $\alpha^{0}$, local parameters ${\left\{\alpha_{i}^{0}\right\}}_{i=1}^{n}$, and dual variables ${\left\{\gamma_{i}^{0}\right\}}_{i=1}^{n}$.2:**for** k=1 to *K* **do**3:    **for** i=1 to *n* **do**4:        **for** t=1 to $t_{\max}$ **do**5:           Construct the local cost function $J(\alpha_{i})$ using Equation (14).6:           Compute the gradient $\nabla J(\alpha_{i}^{\left(t\right)})$ using Equation (16).7:           Compute the Hessian matrix $H(\alpha_{i}^{\left(t\right)})$ using Equation (17).8:           Update the step size $a_{t}$ and local parameter $\alpha_{i}^{\left(t+1\right)}$ using Equation (15).9:          **end for**10:        Set the *k*-th iteration local parameter as $\alpha_{i}^{k}=\alpha_{i}^{\left(t_{\max}\right)}$.11:    **end for**12:    Update the global parameter $\alpha^{k}$ using Equation (18).13:    **for** i=1 to *n* **do**14:        Update the dual variable $\gamma_{i}^{k}$ using Equation (19).15:    **end for**16:**end for**17:**return** Final global model parameter $\alpha^{K}$.

During each iteration, local computations are executed in parallel across all edge nodes. Communication is confined exclusively to parameter exchanges between nodes and the central server. This decentralized architecture simultaneously reduces computational load on the central server and preserves data privacy by retaining raw data locally at edge nodes. Critically, theoretical convergence guarantees from ADMM are integrated with Newton’s method for efficient local optimization, enabling the framework to achieve both global model consistency and practical training efficiency. These characteristics render it ideally suited for federated learning scenarios under privacy constraints.

## 4. DLGP Algorithm

The ADMM-based distributed logistic regression framework decomposes centralized optimization tasks into *n* subproblems, enabling collaborative training across *n* edge nodes. While this architecture reduces network bandwidth requirements and offers partial protection for raw user data, privacy risks persist during parameter transmission where adversaries could potentially reconstruct training data through analysis of historical model parameters uploaded to the central server. To address this vulnerability, a differential privacy mechanism that injects noise into local parameter updates is introduced, thereby enhancing the algorithm’s privacy guarantees. The injected noise strategically increases the entropy of the transmitted parameters, making it information-theoretically harder for adversaries to reconstruct sensitive training data from the observed model updates. The proposed DLGP algorithm is detailed below.

### 4.1. $\ell_{2}$-Sensitivity Analysis

For (ϵ,δ)-differential privacy implementation via a Gaussian mechanism, sensitivity analysis must first be conducted to quantify variations in local parameter updates resulting from minor input dataset modifications. In the DLGP algorithm, the $\ell_{2}$-sensitivity of local parameter $\alpha_{i}^{k}$ is analyzed, defined as the maximum $\ell_{2}$-norm difference between parameter updates derived from adjacent datasets differing by exactly one sample.

Consider two adjacent local datasets $D_{i}$ and $D_{i}^{\prime }$ at edge node *i*, where $|D_{i}▵D_{i}^{\prime }|=1$ (i.e., the symmetric difference contains exactly one sample). Without loss of generality, the discrepancy is assumed to occur in the first sample. The local parameters computed from $D_{i}$ and $D_{i}^{\prime }$ are denoted as $\alpha_{i,D_{i}}^{k}$ and $\alpha_{i,D_{i}^{\prime }}^{k}$, respectively, which are obtained by minimizing slightly different objective functions

$$\begin{array}{ccc} \alpha_{i,D_{i}}^{k} & = & \arg\min_{\alpha_{i}}J\left(\alpha_{i}\right),\\ \alpha_{i,D_{i}^{\prime }}^{k} & = & \arg\min_{\alpha_{i}}J\left(\alpha_{i}\right)+G\left(\alpha_{i}\right), \end{array}$$

where $J\left(\alpha_{i}\right)=L_{i}\left(\alpha_{i},\alpha ,\gamma_{i}\right)$ represents the local cost function from Section 3, and $G(\alpha_{i})$ captures the logistic loss difference introduced by the modified sample

(20) $$G\left(\alpha_{i}\right)=\frac{1}{m_{i}}\log\left(1+\exp\left(-y_{i1}^{\prime }\alpha_{i}^{T}x_{i1}^{\prime }\right)\right)-\frac{1}{m_{i}}\log\left(1+\exp\left(-y_{i1}\alpha_{i}^{T}x_{i1}\right)\right).\;$$

From the smoothness properties of the objective functions, the gradients at their respective minima satisfy $\nabla J(\alpha_{i,D_{i}}^{k})=0$ and $\nabla J\left(\alpha_{i,D_{i}^{\prime }}^{k}\right)+\nabla G\left(\alpha_{i,D_{i}^{\prime }}^{k}\right)=0$. Given the (λ/n+μ)-strong convexity of $J(\alpha_{i})$, the inequality

(21) $${\left(\nabla J\left(\alpha_{i,D_{i}}^{k}\right)-\nabla J\left(\alpha_{i,D_{i}^{\prime }}^{k}\right)\right)}^{T}\left(\alpha_{i,D_{i}}^{k}-\alpha_{i,D_{i}^{\prime }}^{k}\right)\geq \left(\frac{\lambda }{n}+\mu \right){∥\alpha_{i,D_{i}}^{k}-\alpha_{i,D_{i}^{\prime }}^{k}∥}_{2}^{2}\;$$

is satisfied. Application of the Cauchy–Schwarz inequality to the left-hand side yields

(22) $${\left(\nabla J\left(\alpha_{i,D_{i}}^{k}\right)-\nabla J\left(\alpha_{i,D_{i}^{\prime }}^{k}\right)\right)}^{T}\left(\alpha_{i,D_{i}}^{k}-\alpha_{i,D_{i}^{\prime }}^{k}\right)\leq ∥\alpha_{i,D_{i}}^{k}-\alpha_{i,D_{i}^{\prime }}^{k}{∥}_{2}∥\nabla G\left(\alpha_{i,D_{i}^{\prime }}^{k}\right){∥}_{2}.$$

Combining Equations (21) and (22) and substituting the expression for $\nabla G(\alpha_{i})$ leads to

(23) $$\begin{array}{ccc} ∥\alpha_{i,D_{i}}^{k}-\alpha_{i,D_{i}^{\prime }}^{k}{∥}_{2} & \leq & \frac{n{∥\frac{-y_{i1}^{\prime }x_{i1}^{\prime }}{1+\exp\left(y_{i1}^{\prime }\alpha_{i}^{T}x_{i1}^{\prime }\right)}-\frac{-y_{i1}x_{i1}}{1+\exp\left(y_{i1}\alpha_{i}^{T}x_{i1}\right)}∥}_{2}}{m_{i}\left(\lambda +\mu n\right)}. \end{array}$$

By the triangle inequality, the numerator term is bounded as

$${∥\frac{-y_{i1}^{\prime }x_{i1}^{\prime }}{1+\exp\left(y_{i1}^{\prime }\alpha_{i}^{T}x_{i1}^{\prime }\right)}-\frac{-y_{i1}x_{i1}}{1+\exp\left(y_{i1}\alpha_{i}^{T}x_{i1}\right)}∥}_{2}\leq {∥\frac{-y_{i1}^{\prime }x_{i1}^{\prime }}{1+\exp\left(y_{i1}^{\prime }\alpha_{i}^{T}x_{i1}^{\prime }\right)}∥}_{2}+{∥\frac{-y_{i1}x_{i1}}{1+\exp\left(y_{i1}\alpha_{i}^{T}x_{i1}\right)}∥}_{2},$$

where each term is bounded by 1 due to sigmoid function properties, resulting in

(24) $${∥\frac{-y_{i1}^{\prime }x_{i1}^{\prime }}{1+\exp\left(y_{i1}^{\prime }\alpha_{i}^{T}x_{i1}^{\prime }\right)}-\frac{-y_{i1}x_{i1}}{1+\exp\left(y_{i1}\alpha_{i}^{T}x_{i1}\right)}∥}_{2}\leq 2.$$

Substituting Equation (24) into Equation (23) gives the $\ell_{2}$-sensitivity of the local parameter update $\alpha_{i}^{k}$ as

(25) $$\max∥\alpha_{i,D_{i}}^{k}-\alpha_{i,D_{i}^{\prime }}^{k}{∥}_{2}=\frac{2}{m_{i}\left(\lambda /n+\mu \right)}.$$

This sensitivity bound quantifies the maximum information leakage that could occur from a single data point modification, providing a foundation for calibrating the noise required to achieve the desired entropy increase in the output distribution.

### 4.2. Dynamic Noise Generation and Perturbation Implementation

**Figure 2:**
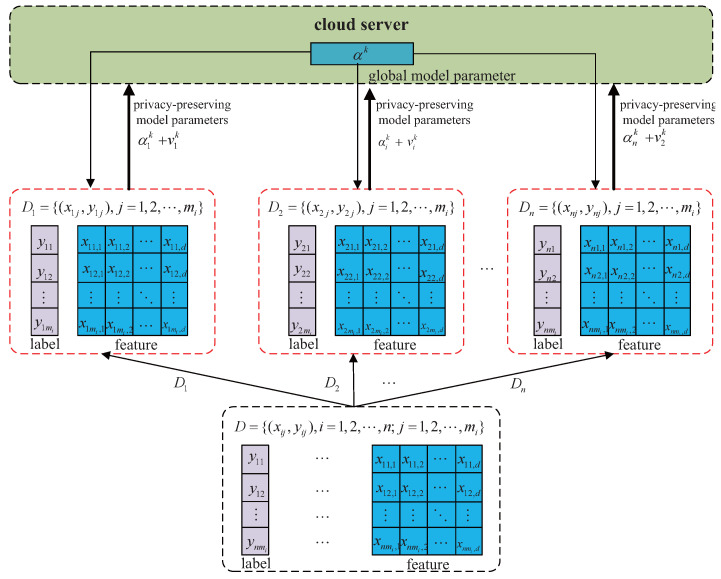
Flow chart of the DLGP algorithm.

The workflow of the DLGP algorithm is illustrated in Figure 2. Computational tasks are distributed by the central server to *n* edge nodes. During the *k*-th iteration, local parameters are computed by each edge node using Newton iterations as described in Section 3. To prevent privacy leakage, calibrated Gaussian noise is injected into these local parameters prior to transmission to the server.

Based on the Gaussian mechanism for differential privacy and the sensitivity result in Equation (25), the noise added to each local parameter must follow a Gaussian distribution $v_{i}^{k}∼N\left(0,\sigma^{2}I_{d}\right)$, where the standard deviation σ is calibrated as

(26) $$\sigma \geq \frac{2\sqrt{2\ln(1.25/\delta )}}{m_{i}\left(\lambda /n+\mu \right)\epsilon },$$

which ensures that the perturbed parameter ${\tilde{\alpha }}_{i}^{k}=\alpha_{i}^{k}+v_{i}^{k}$ satisfies (ϵ,δ)-differential privacy for each update step. The Gaussian distribution is chosen specifically because it represents the maximum entropy distribution for a given variance, meaning it provides the greatest amount of uncertainty for a given noise power constraint. This optimal entropy property makes Gaussian noise particularly effective for obscuring the original parameter values while minimizing the impact on utility.

Following privacy protection implementation across all edge nodes, perturbed values ${\left\{{\tilde{\alpha }}_{i}^{k}\right\}}_{i=1}^{n}$ are uploaded to the central server for global aggregation. The global parameter update rule is adjusted for perturbed local parameters as

(27) $$\alpha^{k}=\frac{1}{n}\sum_{i=1}^{n}{\tilde{\alpha }}_{i}^{k}-\frac{1}{n}\sum_{i=1}^{n}\frac{\gamma_{i}^{k-1}}{\mu }.$$

Subsequently, dual variables are updated by each edge node using perturbed local and aggregated global parameters

(28) $$\gamma_{i}^{k}=\gamma_{i}^{k-1}-\mu \left({\tilde{\alpha }}_{i}^{k}-\alpha^{k}\right).$$

Updated dual variables and global parameters are then utilized for the (k+1)-th iteration of local parameter optimization. This process continues until *K* total iterations are completed, at which point final global parameter $\alpha^{K}$ is returned. The iterative noise injection creates a cumulative privacy protection effect, where each round of perturbation further increases the uncertainty about the original training data, effectively implementing a form of progressive information obfuscation throughout the learning process. The complete DLGP algorithm is summarized in Algorithm 2.
**Algorithm 2** Pseudocode for the DLGP algorithm**Require:** Local datasets $D={\left\{x_{ij},y_{ij}\right\}}_{i=1,\ldots ,n;j=1,\ldots ,m_{i}}$.**Ensure:** Final global model parameter $\alpha^{K}$.1:Initialize $\alpha^{0}$, ${\left\{\alpha_{i}^{0}\right\}}_{i=1}^{n}$, and ${\left\{\gamma_{i}^{0}\right\}}_{i=1}^{n}$.2:**for** k=1 to *K* **do**3:    **for** i=1 to *n* **do**4:        **for** t=1 to $t_{\max}$ **do**5:           Construct the local cost function $J(\alpha_{i})$ using Equation (14).6:           Update the gradient $\nabla J(\alpha_{i}^{\left(t\right)})$ using Equation (16).7:           Update the Hessian matrix $H(\alpha_{i}^{\left(t\right)})$ using Equation (17).8:           Update the step size $a_{t}$ and local parameter $\alpha_{i}^{\left(t+1\right)}$ using Equation (15).9:          **end for**10:        Set the local parameter $\alpha_{i}^{k}=\alpha_{i}^{\left(t_{\max}\right)}$.11:        Compute the sensitivity using Equation (25).12:        Calculate the Gaussian noise standard deviation σ using Equation (26).13:        Generate Gaussian noise $v_{i}^{k}∼N\left(0,\sigma^{2}I_{d}\right)$.14:        Apply perturbation: ${\tilde{\alpha }}_{i}^{k}=\alpha_{i}^{k}+v_{i}^{k}$.15:    **end for**16:    Update the global parameter $\alpha^{k}$ using Equation (27).17:    **for** i=1 to *n* **do**18:        Update the dual variable $\gamma_{i}^{k}$ using Equation (28).19:    **end for**20:**end for**21:**return** Final global model parameter $\alpha^{K}$.

In practical deployments, the selection of the privacy budget ϵ critically influences the trade-off between privacy guarantees and model utility. A smaller ϵ value enforces stronger privacy protection by increasing the noise scale according to Equation (26), which can potentially degrade model accuracy by obscuring the true parameter updates. However, for larger datasets, the impact of noise is mitigated due to the reduced $\ell_{2}$-sensitivity per data point, as sensitivity is inversely proportional to the local dataset size $m_{i}$. This implies that in data-rich environments, even stringent privacy settings can be achieved without substantial loss in utility, as the model benefits from greater statistical stability.

### 4.3. Computational Complexity Analysis

The computational complexity of DLGP primarily stems from local parameter updates using Newton’s method, global parameter aggregation, dual variable updates, and the additional privacy protection mechanism. Consider a federated system with *n* edge nodes, each maintaining a local dataset of size $m_{i}$ (assumed equal to *m* for simplicity), feature dimension *d*, ADMM iteration count *K*, and maximum Newton iterations $t_{\max}$ per ADMM round.

The local parameter update represents the most computationally intensive component of the algorithm. At each edge node during every ADMM iteration, Newton’s method is employed to minimize the local augmented Lagrangian function. The gradient computation according to Equation (16) requires evaluating the logistic loss gradient, regularization term, and constraint penalties across all *m* local samples, resulting in O(md) complexity. The Hessian matrix calculation following Equation (17) involves computing the sum of outer products for all samples, yielding $O(md^{2})$ complexity. The Newton update step in Equation (15) necessitates solving a linear system through matrix inversion, which incurs $O(d^{3})$ complexity. With $t_{\max}$ Newton iterations per ADMM round, the total local computation complexity per node becomes $O(t_{\max}\left(md^{2}+d^{3}\right))$.

The privacy protection mechanism introduces additional computational overhead that must be quantified. The $\ell_{2}$-sensitivity calculation derived in Equation (25) involves a closed-form expression computable in O(1) time. Gaussian noise generation according to Equation (26) requires sampling *d* independent random values, resulting in O(d) complexity. Although these privacy operations add incremental costs, they are negligible compared to the local optimization overhead.

Global aggregation and dual variable updates contribute moderately to the overall computational burden. The server-side global parameter aggregation in Equation (27) computes the average of *n* perturbed local parameters, requiring O(nd) operations. The dual variable updates at each edge node following Equation (28) involve simple vector operations with O(d) complexity per node, totaling O(nd) across all nodes.

The overall computational complexity of DLGP across *K* ADMM iterations combines all these components. The dominant factor is the local Newton optimization, which scales with $O(Knt_{\max}\left(md^{2}+d^{3}\right))$. The privacy protection and coordination operations contribute O(Knd), which becomes negligible for practical problems where $d^{2}$ or $d^{3}$ dominates. The specific complexity characteristics depend on the relationship between feature dimension *d* and sample size *m*. When d≪m, the $O(md^{2})$ term dominates, making complexity linear in sample size. When *d* is large (d∼m or d>m), the $O(d^{3})$ term from matrix inversion becomes predominant.

Communication overhead represents another critical aspect of distributed algorithm performance. In each ADMM iteration, every edge node uploads a perturbed *d*-dimensional parameter vector and downloads the global *d*-dimensional parameter, resulting in O(nd) communication per round. The total communication overhead over *K* iterations is O(Knd), which is identical to the baseline ADMM algorithm without privacy protection. This demonstrates that the privacy mechanism does not increase communication overhead.

Therefore, compared to the standard ADMM framework, DLGP introduces minimal additional computational burden. The privacy operations add only O(Knd) to the overall complexity, which is dominated by the $O(Knt_{\max}\left(md^{2}+d^{3}\right))$ local optimization costs.

The computational complexity analysis reveals that the DLGP algorithm maintains practical efficiency for edge computing environments. As summarized in Table 2, the dominant computational cost stems from the local Newton optimization, specifically the Hessian computation ($O(md^{2})$) and matrix inversion ($O(d^{3})$) operations. The privacy protection mechanism introduces only linear overhead O(nd), which becomes negligible compared to the polynomial terms in the local optimization. Importantly, the communication overhead remains at O(nd) per iteration, involving only the transmission of parameter vectors between edge nodes and the central server. The favorable complexity profile ensures the algorithm’s scalability while maintaining rigorous privacy guarantees through differential privacy.

## 5. Privacy Analysis of the DLGP Algorithm

This section presents a rigorous proof of the differential privacy guarantee for the DLGP algorithm, establishing the theoretical foundation for its privacy-preserving capabilities. Specifically, we analyze the sensitivity of local parameter updates within the ADMM framework, derive the precise noise magnitude required for Gaussian perturbation, and formally verify that the proposed mechanism satisfies (ϵ,δ)-differential privacy. This theoretical analysis not only validates the algorithm’s privacy guarantees but also provides quantifiable bounds for balancing privacy protection and model utility in practical deployments. The privacy proof essentially establishes an upper bound on the information leakage that could occur through the model updates, ensuring that the algorithm preserves the confidentiality of individual data points while maintaining useful aggregate statistics.

**Theorem** **1.** *Given privacy budget ϵ∈(0,1) and failure probability δ∈(0,1), let $v_{i}^{k}$ be Gaussian noise with mean 0 and variance $\sigma^{2}$ satisfying*

$$\sigma \geq \frac{2\sqrt{2\ln(1.25/\delta )}}{m_{i}\left(\lambda /n+\mu \right)\epsilon }.$$

*The Gaussian mechanism guarantees (ϵ,δ)-differential privacy such that for any adjacent datasets $D_{i}$ and $D_{i}^{\prime }$, and any output ${\tilde{\alpha }}_{i}^{k}$, the inequality*

(29) $$\mathrm{Pr}\left[{\tilde{\alpha }}_{i}^{k}|D_{i}\right]\leq \exp\left(\epsilon \right)\cdot \mathrm{Pr}\left[{\tilde{\alpha }}_{i}^{k}|D_{i}^{\prime }\right]+\delta$$

*holds.*

**Proof.**  Let ${\tilde{\alpha }}_{i}^{k}$, $v_{i}^{k}$, and ${v^{\prime }}_{i}^{k}$ represent arbitrary components of ${\tilde{\alpha }}_{i}^{k}$, $v_{i}^{k}$, and ${v^{\prime }}_{i}^{k}$, respectively, where both $v_{i}^{k}$ and ${v^{\prime }}_{i}^{k}$ are Gaussian noise with mean 0 and variance $\sigma^{2}$. The privacy loss for output ${\tilde{\alpha }}_{i}^{k}$ is expressed as

(30) $$\begin{array}{cc} \left|\ln\frac{\mathrm{Pr}[{\tilde{\alpha }}_{i}^{k}|D_{i}]}{\mathrm{Pr}[{\tilde{\alpha }}_{i}^{k}|D_{i}^{\prime }]}\right| & =|\ln\frac{\mathrm{Pr}[{\tilde{\alpha }}_{i}^{k}|D_{i}]}{\mathrm{Pr}[{\tilde{\alpha }}_{i}^{k}|D_{i}^{\prime }]}|\\ & =|\ln\frac{\mathrm{Pr}[{\tilde{\alpha }}_{i,D_{i}}^{k}=\alpha_{i,D_{i}}^{k}+v_{i}^{k}]}{\mathrm{Pr}[{\tilde{\alpha }}_{i,D_{i}}^{k}=\alpha_{i,{D^{\prime }}_{i}}^{k}+{v^{\prime }}_{i}^{k}]}|\\ & =|\ln\frac{\mathrm{Pr}[v_{i}^{k}]}{\mathrm{Pr}[{v^{\prime }}_{i}^{k}]}|\\ & =\left|\frac{1}{2\sigma^{2}}\left(|v_{i}^{k}{|}^{2}-{\left|{v^{\prime }}_{i}^{k}\right|}^{2}\right)\right|\\ & =\left|\frac{1}{2\sigma^{2}}\left(|v_{i}^{k}{|}^{2}-{\left|v_{i}^{k}+\alpha_{i,D_{i}}^{k}-\alpha_{i,D_{i}^{\prime }}^{k}\right|}^{2}\right)\right|\\ & =\left|\frac{1}{2\sigma^{2}}\left(2v_{i}^{k}|\alpha_{i,D_{i}}^{k}-\alpha_{i,D_{i}^{\prime }}^{k}\left|+\right|\alpha_{i,D_{i}}^{k}-\alpha_{i,D_{i}^{\prime }}^{k}{|}^{2}\right)\right|. \end{array}$$

To bound the privacy loss within the privacy budget ϵ, the condition

(31) $$\begin{array}{cc} \epsilon & \geq |\ln\frac{\mathrm{Pr}[{\tilde{\alpha }}_{i}^{k}|D_{i}]}{\mathrm{Pr}[{\tilde{\alpha }}_{i}^{k}|D_{i}^{\prime }]}|\\ & \geq \left|\frac{1}{2\sigma^{2}}\left(2v_{i}^{k}|\alpha_{i,D_{i}}^{k}-\alpha_{i,D_{i}^{\prime }}^{k}\left|+\right|\alpha_{i,D_{i}}^{k}-\alpha_{i,D_{i}^{\prime }}^{k}{|}^{2}\right)\right| \end{array}$$

must be satisfied. Since $|\alpha_{i,D_{i}}^{k}-\alpha_{i,D_{i}^{\prime }}^{k}|\leq ∥\alpha_{i,D_{i}}^{k}-\alpha_{i,D_{i}^{\prime }}^{k}{∥}_{2}$, substitution of the sensitivity result from Equation (25) yields the bound

$$|v_{i}^{k}|\leq \frac{\epsilon \sigma^{2}m_{i}\left(\lambda +\mu n\right)}{2n}-\frac{n}{m_{i}\left(\lambda +\mu n\right)}.$$

For notational convenience, define the threshold value

(32) $$z=\frac{\epsilon \sigma^{2}m_{i}\left(\lambda +\mu n\right)}{2n}-\frac{n}{m_{i}\left(\lambda +\mu n\right)}.$$

The ϵ-differential privacy requirement is satisfied when $|v_{i}^{k}|\leq z$, as this condition ensures $\ln\left(\frac{\mathrm{Pr}[{\tilde{\alpha }}_{i}^{k}|D_{i}]}{\mathrm{Pr}[{\tilde{\alpha }}_{i}^{k}|D_{i}^{\prime }]}\right)\leq \epsilon .$ For scenarios where $|v_{i}^{k}|>z$, privacy leakage must be bounded by δ, necessitating $\mathrm{Pr}[|v_{i}^{k}|>z]\leq \delta .$ By symmetry of the Gaussian probability density function, the one-sided tail probability is constrained as

(33) $$\mathrm{Pr}\left[v_{i}^{k}>z\right]\leq \frac{\delta }{2}.$$

Using the probability density function of the Gaussian distribution, the left-hand side of Equation (33) can be rewritten as

(34) $$\begin{array}{ccc} \mathrm{Pr}[v_{i}^{k}>z] & = & \frac{1}{\sqrt{2\pi }\sigma }\int_{z}^{\infty }\exp\left(-\frac{{\left(v_{i}^{k}\right)}^{2}}{2\sigma^{2}}\right)dv_{i}^{k}\\ & < & \frac{1}{\sqrt{2\pi }\sigma }\int_{z}^{\infty }\frac{v_{i}^{k}}{z}\exp\left(-\frac{{\left(v_{i}^{k}\right)}^{2}}{2\sigma^{2}}\right)dv_{i}^{k}\\ & = & \frac{\sigma }{\sqrt{2\pi }z}\exp\left(-\frac{z^{2}}{2\sigma^{2}}\right). \end{array}$$

Substituting Equation (34) into Equation (33) gives

(35) $$\begin{array}{ccc} \frac{\sigma }{\sqrt{2\pi }z}\exp\left(-\frac{z^{2}}{2\sigma^{2}}\right) & < & \frac{\delta }{2}\\ \frac{z}{\sigma }\exp\left(\frac{z^{2}}{2\sigma^{2}}\right) & > & \frac{2}{\sqrt{2\pi }\delta }\\ \ln\frac{z}{\sigma }+\frac{z^{2}}{2\sigma^{2}} & > & \ln\frac{2}{\sqrt{2\pi }\delta }. \end{array}$$

To determine the required standard deviation σ for the Gaussian mechanism, sufficient conditions for Equation (35) are established through the inequalities

(36) $$\begin{array}{cc} \ln\frac{z}{\sigma } & \geq 0, \end{array}$$

(37) $$\begin{array}{cc} \frac{z^{2}}{2\sigma^{2}} & >\ln\frac{2}{\sqrt{2\pi }\delta }. \end{array}$$

Analysis begins with Equation (36) where

(38) $$\sigma =\frac{2nc}{m_{i}\left(\lambda +\mu n\right)\epsilon }$$

with c≥1. Substitution into Equation (32) yields

(39) $$\frac{z}{\sigma }=c-\frac{\epsilon }{2c}.$$

Since ϵ<1 and c≥1, Equation (36) becomes

$$\ln\frac{z}{\sigma }=\ln\left(c-\frac{\epsilon }{2c}\right)\geq \ln\left(c-\frac{1}{2}\right),$$

which implies c≥3/2 when ln(z/σ)≥0. Next, squaring both sides of Equation (39) gives

(40) $$\begin{array}{ccc} {\left(\frac{z}{\sigma }\right)}^{2} & = & {\left(c-\frac{\epsilon }{2c}\right)}^{2}\\ & = & c^{2}-\epsilon +\frac{\epsilon^{2}}{4c^{2}}\\ & > & c^{2}-\frac{8}{9}. \end{array}$$

Since the function $F\left(c\right)=c^{2}-\epsilon +\frac{\epsilon^{2}}{4c^{2}}$ satisfies ∇F(c)>0, Equation (40) holds. Substituting Equation (40) into Equation (37) yields

$$\begin{array}{cc} {\left(\frac{z}{\sigma }\right)}^{2} & >2\ln\frac{2}{\sqrt{2\pi }\delta }\\ c^{2}-\frac{8}{9} & >2\ln\frac{2}{\sqrt{2\pi }\delta }\\ c^{2} & >\frac{8}{9}+\ln\frac{2}{\pi }+2\ln\frac{1}{\delta }\\ c^{2} & >\ln\exp\left(\frac{8}{9}\right)+\ln\frac{2}{\pi }+2\ln\frac{1}{\delta }\\ c^{2} & >2\ln\frac{1.25}{\delta }, \end{array}$$

which sufficiently ensures Equation (35) holds. Consequently, for $\sigma \geq 2n\sqrt{2\ln(1.25/\delta )}/\left(m_{i}\left(\lambda +\mu n\right)\epsilon \right)$, the probability

$$\mathrm{Pr}\left[v_{i}^{k}>\frac{\epsilon \sigma^{2}m_{i}\left(\lambda +\mu n\right)}{2n}-\frac{n}{m_{i}\left(\lambda +\mu n\right)}\right]\leq \frac{\delta }{2}$$

is established, and similarly

$$\mathrm{Pr}\left[|v_{i}^{k}|>\frac{\epsilon \sigma^{2}m_{i}\left(\lambda +\mu n\right)}{2n}-\frac{n}{m_{i}\left(\lambda +\mu n\right)}\right]\leq \delta .$$

Partition R into two subsets: $R=R_{1}\cup R_{2}$, where

$$R_{1}=\left\{v_{i}^{k}\in R|\left|v_{i}^{k}\right|\leq \frac{\epsilon \sigma^{2}m_{i}\left(\lambda +\mu n\right)}{2n}-\frac{n}{m_{i}\left(\lambda +\mu n\right)}\right\}$$

and

$$R_{2}=\left\{v_{i}^{k}\in R|\left|v_{i}^{k}\right|>\frac{\epsilon \sigma^{2}m_{i}\left(\lambda +\mu n\right)}{2n}-\frac{n}{m_{i}\left(\lambda +\mu n\right)}\right\}.$$

For any subset O⊆R, define $O_{1}=\left\{\alpha_{i,D_{i}}^{k}+v_{i}^{k}|v_{i}^{k}\in R_{1}\right\}$ and $O_{2}=\left\{\alpha_{i,D_{i}}^{k}+v_{i}^{k}|v_{i}^{k}\in R_{2}\right\}$. The probability of any point in *O* is bounded as

$$\begin{array}{cc} \mathrm{Pr}[{\tilde{\alpha }}_{i}^{k}|D_{i}] & =\mathrm{Pr}\left[\alpha_{i,D_{i}}^{k}+v_{i}^{k}:v_{i}^{k}\in O_{1}\right]+\mathrm{Pr}\left[\alpha_{i,D_{i}}^{k}+v_{i}^{k}:v_{i}^{k}\in O_{2}\right]\\ \; & \leq \mathrm{Pr}[\alpha_{i,D_{i}}^{k}+v_{i}^{k}:v_{i}^{k}\in O_{1}]+\delta \\ \; & \leq \exp\left(\epsilon \right)\cdot \mathrm{Pr}\left[{\tilde{\alpha }}_{i}^{k}|D_{i}^{\prime }\right]+\delta , \end{array}$$

satisfying (ϵ,δ)-differential privacy and completing the proof of Theorem 1. □

## 6. Experimental Simulation

This section presents experimental results validating the performance of the proposed DLGP algorithm. The privacy–accuracy trade-off, convergence behavior, and parameter sensitivity are evaluated using a real-world occupancy detection dataset, with comparisons against state-of-the-art methods. All experiments are conducted under controlled conditions to ensure reproducibility, with key findings analyzed in conjunction with theoretical results from previous sections.

### 6.1. Dataset and Experimental Setup

The experimental evaluation was performed on the Room Occupancy Estimation dataset from UCI Machine Learning Repository [28], containing 10,129 time-series samples collected from a controlled smart building room. Each sample consists of 18 multivariate features (e.g., temperature, light, sound, CO_2_, digital passive infrared) and a corresponding occupancy label. The dataset was preprocessed with features max-normalized to [0, 1], followed by $\ell_{2}$ normalization of each sample to unit norm. The original labels reflect the number of occupants in the room during the 4-day controlled data collection period, ranging from 0 to 3. To meet the model’s input specification, we recoded the labels by assigning a value of −1 to samples with zero occupants and +1 to those with one to three occupants. For each experiment, 8000 samples were used as the training set, with the remaining 2129 samples serving as the test set. Simulations were implemented in MATLAB R2023b and executed on a machine equipped with a 3.4 GHz processor and 8 GB of RAM. The current experimental setup assumes homogeneous data distribution across all edge nodes and static client participation throughout the training process. Unless otherwise specified, the default parameters are summarized in Table 3.

To validate the effectiveness of the DLGP algorithm, comparative experiments were conducted with five baseline methods, including a centralized strategy, ADMM, Distributed Logistic Variable Perturbation (DLVP) algorithm [24], Differentially Private Stochastic Gradient Descent (DPSGD) [29], and Differentially Private Per-Sample Adaptive Clipping Federated Learning (DP-PSAC-FL) [30]. For DPSGD and DP-PSAC-FL, the learning rate was set to 0.1. Additionally, in DP-PSAC-FL, the stability constant was configured as 0.1. The evaluation employed two key metrics: classification accuracy and empirical loss.

The empirical loss function is defined as

$$\frac{1}{n}\sum_{i=1}^{n}\sum_{j=1}^{m_{i}}\frac{1}{m_{i}}\log\left(1+\exp\left(-y_{ij}{\tilde{\alpha }}_{i}^{T}x_{ij}\right)\right),$$

where ${\tilde{\alpha }}_{i}$ denotes the perturbed local parameter vector at edge node *i*, $x_{ij}$ represents the *j*-th feature vector at node *i*, and $y_{ij}$ is the corresponding label.

### 6.2. Performance Analysis with Varying Number of Nodes

To evaluate the impact of edge node quantity on algorithm performance, both empirical loss and accuracy were measured under different node configurations n∈{50,80,100,200} and privacy budgets ϵ∈{0.5,0.9}. This dual-metric evaluation addresses the distinction between data fitting and classification performance while validating consistency between experimental results and theoretical conclusions from Theorem 1.

**Figure 3:**
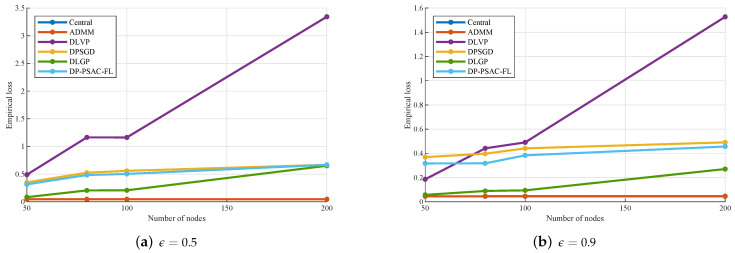
Empirical loss with different numbers of edge nodes.

As presented in Table 4 and Figure 3, all privacy-preserving algorithms (DLGP, DLVP, DPSGD, and DP-PSAC-FL) exhibit a consistent upward trend in empirical loss with increasing *n*. The centralized strategy, which is inherently independent of node quantity, maintains identical loss values across all *n* configurations. ADMM, as a distributed non-privacy-preserving baseline, also shows negligible variations in loss. Under ϵ=0.5, the empirical loss of DLGP increases from 0.0840 at n=50 to 0.6509 at n=200, a range substantially lower than the corresponding increases observed for DLVP (from 0.4897 to 3.3431), DPSGD (from 0.3502 to 0.6685), and DP-PSAC-FL (from 0.3150 to 0.6679). A similar behavior is observed for ϵ=0.9, with DLGP consistently outperforming all comparative privacy-preserving algorithms. Taking the default number of nodes n=100 as an example, DLGP reduces the empirical loss to 0.0943, which is approximately 80.8% lower than DLVP (0.4906), around 78.6% lower than DPSGD (0.4415), and about 75.4% lower than DP-PSAC-FL (0.3837). This observation aligns with Theorem 1, which stipulates that noise intensity is proportional to $1/m_{i}$. An increase in *n* reduces $m_{i}$, necessitating the injection of additional noise to sustain privacy protection and thereby leading to elevated empirical loss.

**Figure 4:**
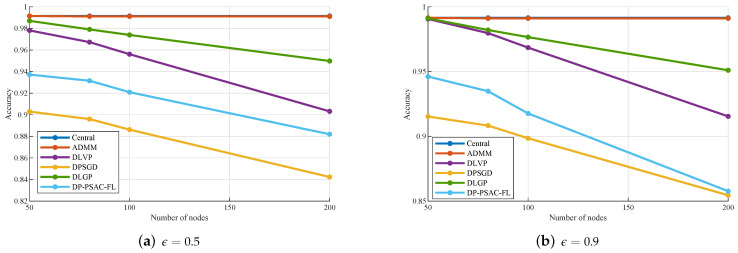
Accuracy with different numbers of edge nodes.

Table 5 and Figure 4 further confirm an inverse relationship between accuracy and node quantity for privacy-preserving methods. Under ϵ=0.5, DLGP maintains high accuracy with a mild degradation from 0.9870 at n=50 to 0.9498 at n=200. In contrast, DLVP, DPSGD, and DP-PSAC-FL undergo more pronounced accuracy declines, falling from 0.9782 to 0.9031, from 0.9029 to 0.8423, and from 0.9372 to 0.8819, respectively. For ϵ=0.9, DLGP’s accuracy decreases slightly from 0.9911 to 0.9510, while maintaining a clear advantage over other privacy-preserving methods. At n=100, its accuracy reaches 0.9766, representing an improvement of 0.81% over DLVP (0.9685), 7.81% over DPSGD (0.8985), and 6.03% over DP-PSAC-FL (0.9177). This keeps DLGP’s performance closer to that of non-privacy-preserving baselines compared to other privacy-aware algorithms. The stable accuracy of the centralized strategy and ADMM confirms that privacy-unconstrained methods are unaffected by variations in node quantity, providing a benchmark for assessing the privacy–performance trade-off of the proposed DLGP and comparative algorithms.

### 6.3. Impact of Privacy Budget ϵ

To provide a more intuitive demonstration of how privacy budget ϵ impacts empirical loss, experimental results for five algorithms (ADMM, DLVP, DPSGD, DP-PSAC-FL, and the proposed DLGP) are analyzed under varying privacy budgets ϵ∈{0.3,0.5,0.7,0.9}, with the number of edge nodes fixed at 100. From the overall experimental trends, all five algorithms show a consistent downward shift in empirical loss as ϵ increases from 0.3 to 0.9, and each algorithm achieves its lowest empirical loss at ϵ=0.9 relative to other privacy levels. This trend aligns with Theorem 1, which specifies that a larger privacy budget corresponds to a smaller noise variance, with reduced noise injection during training minimizing deviations between model predictions and true labels and in turn lowering empirical loss.

**Figure 5:**
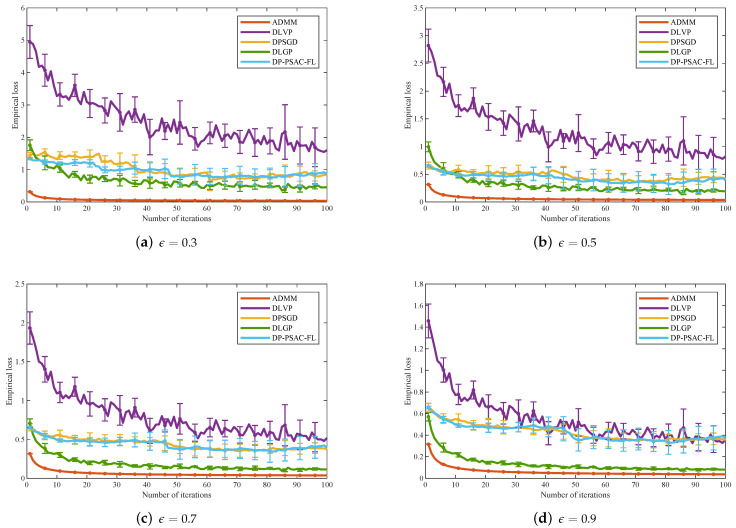
Empirical loss under different privacy budgets.

As illustrated in Figure 5d, when ϵ=0.9, ADMM maintains the lowest empirical loss among all algorithms and exhibits nearly no error fluctuations. This phenomenon is attributed to ADMM’s nature as a distributed non-privacy-preserving algorithm, which does not introduce any privacy-related noise into intermediate variable transmission or local parameter updates during training. Its empirical loss is thus only determined by model fitting performance, free from noise interference, leading to both minimal and stable loss values. Among the privacy-preserving algorithms (DLVP, DPSGD, DLGP, DP-PSAC-FL), the proposed DLGP outperforms DLVP and DPSGD significantly at ϵ=0.9. DLGP outperforms DLVP in accuracy because the Gaussian mechanism introduces less noise than the Laplace mechanism employed by DLVP. Furthermore, DLGP also surpasses both DPSGD and DP-PSAC-FL, as its design mitigates noise accumulation during training. In contrast, the stochastic gradient-based perturbation mechanisms used in DPSGD and DP-PSAC-FL are more sensitive to gradient fluctuations, leading to higher empirical loss.

To validate the computational efficiency of the proposed DLGP algorithm, we conducted runtime simulations comparing it with three baseline approaches: ADMM, DLVP, DPSGD, and DP-PSAC-FL. Each algorithm was executed five times under identical experimental conditions, and the average computation times were recorded. As shown in Table 6, DLGP achieved a competitive runtime of 2.1555 s, which is comparable to the standard ADMM approach (2.0768 s) while providing enhanced privacy guarantees. The DLVP algorithm exhibited the longest computation time at 2.2812 s, while DPSGD and DP-PSAC-FL demonstrated significantly faster execution at 0.2471 s and 0.3158 s, respectively, due to their simpler optimization frameworks. These results confirm that the additional privacy protection mechanisms in DLGP introduce only modest computational overhead, making it practically viable for real-world federated learning applications where both efficiency and privacy are critical considerations.

### 6.4. Sensitivity to Penalty Parameter

**Figure 6:**
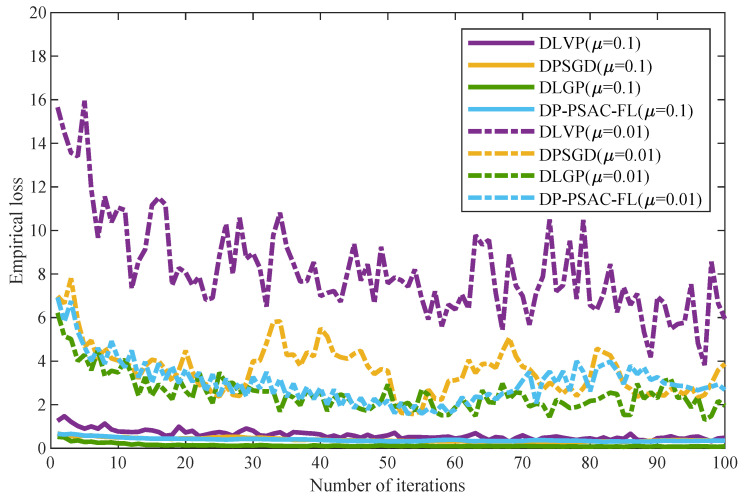
Empirical loss under different penalty parameters.

The impact of the penalty parameter μ on model training performance is analyzed in Figure 6, which displays the empirical loss curves of three privacy-preserving algorithms for μ∈{0.01,0.1}. All algorithms exhibit consistently higher empirical loss at μ=0.01 than at μ=0.1. As iterations increase, their empirical loss generally decreases, but the curves for μ=0.01 consistently remain above those for μ=0.1.

This trend aligns with Theorem 1, which states noise level is inversely proportional to μ. A smaller penalty parameter (μ=0.01) leads to more noise injected during training, disrupting parameter update accuracy and ultimately increasing empirical loss. Among four algorithms, DLGP maintains the lowest empirical loss across both μ settings. Its advantage is particularly prominent under μ=0.01, and even when iterations reach 100, its empirical loss remains lower than that of DLVP, DPSGD, and DP-PSAC-FL. This confirms DLGP possesses stronger adaptability to ADMM penalty parameter variations and sustains superior training performance even in scenarios with more injected noise.

### 6.5. Impact of Regularization Parameter

**Figure 7:**
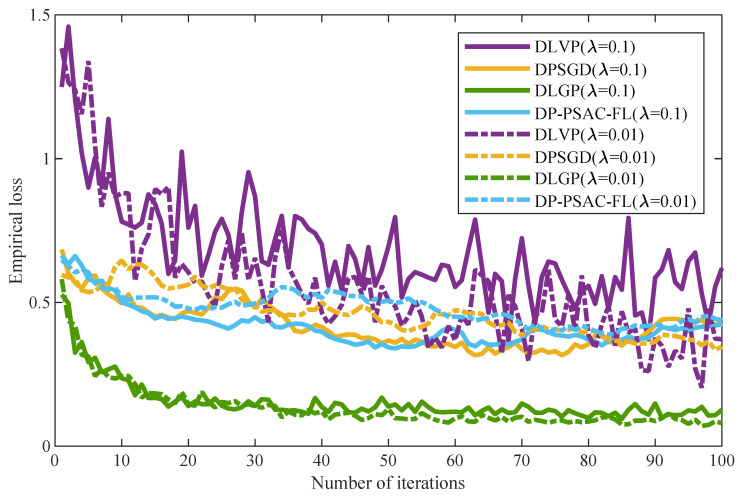
Empirical loss under different regularization parameters.

The influence of the regularization parameter λ on model training performance is evaluated in Figure 7, which depicts the empirical loss curves of DLVP, DPSGD, and DP-PSAC-FL for λ∈{0.01,0.1}. All algorithms exhibit a consistent decline in empirical loss as iterations progress. Notably, the difference in loss between the two λ values is marginal throughout the training process. Among the four methods, DLGP achieves the lowest empirical loss under both λ settings, followed by DLVP and DP-PSAC-FL, while DPSGD consistently yields the highest loss.

This observation aligns with Equation (26), where the impact of λ on noise level is scaled by 1/n. With the default number of edge nodes n=100, the scaling effect weakens the influence of λ on empirical loss, resulting in the minor differences between the loss curves of different λ values observed in the figure. This further confirms that the regularization parameter λ exerts a weaker influence on model empirical loss compared to the ADMM penalty parameter, and the proposed DLGP remains robust to variations in λ while maintaining superior performance.

Based on Figure 3, Figure 4, Figure 5, Figure 6 and Figure 7 and Table 4 and Table 5, we can draw the following conclusions.

1. As the number of edge nodes increases, the empirical loss of privacy-preserving algorithms (DLGP, DLVP, DPSGD, and DP-PSAC-FL) rises, whereas non-private baselines remain stable. Across all node counts, DLGP consistently outperforms other privacy-preserving algorithms in both empirical loss and accuracy, demonstrating superior scalability.
2. Figure 5 demonstrates that empirical loss decreases with increasing privacy budget for all algorithms, with DLGP maintaining lower loss values compared to DLVP, DPSGD, and DP-PSAC-FL across all tested privacy levels.
3. Figure 6 reveals that empirical loss increases with decreasing penalty parameters for all algorithms. The superiority of DLGP over DLVP, DPSGD, and DP-PSAC-FL becomes more pronounced as the penalty parameter decreases.
4. Figure 7 indicates that the regularization parameter has a relatively minor impact on the empirical loss of all privacy-preserving algorithms.

## 7. Conclusions

This paper develops the DLGP algorithm, which integrates ADMM with differential privacy mechanisms to address privacy–utility trade-offs in federated learning scenarios. Theoretical analysis establishes bounded $\ell_{2}$-sensitivity of local parameter updates, enabling rigorous (ϵ,δ)-differential privacy guarantees through calibrated Gaussian noise injection. Experimental evaluations on the Room Occupancy Estimation dataset from the UCI Repository demonstrate DLGP’s superior performance with quantifiable metrics. Under various configurations, DLGP achieves substantially lower empirical loss and higher classification accuracy compared to all baseline methods. This superiority persists across different configurations, with DLGP maintaining stronger robustness to variations in penalty parameter μ and regularization parameter λ than baseline algorithms while upholding formal (ϵ,δ)-differential privacy guarantees.

While the proposed DLGP framework provides formal privacy guarantees for federated logistic regression, its evaluation is limited to the IID data setting. Future work will focus on developing personalized variants of the model through client-specific regularization techniques and exploring robust optimization methods to enhance performance under non-IID data distributions while maintaining the established differential privacy guarantees.

## Figures and Tables

**Table 1:** Key symbols and their descriptions.

Symbol	Definition
$x_{ij}$	Data feature vector
$y_{i}$	Data label
λ	Regularization parameter
α	Global model parameter vector
$\alpha_{i}$	Local model parameter vector at edge node *i*
$\gamma_{i}$	Dual variable at edge node *i*
μ	Penalty parameter
$\alpha_{i}^{k}$	Local parameter vector at edge node *i* in iteration *k*
${\tilde{\alpha }}_{i}^{k}$	Privacy-preserving version of $\alpha_{i}^{k}$
$\gamma_{i}^{k}$	Dual variable at edge node *i* in iteration *k*
$\alpha^{k}$	Global model parameter vector in iteration *k*
$v_{i}^{k}$	Sampled noise at edge node *i* in iteration *k*
σ	Standard deviation of the Gaussian mechanism

**Table 2:** Computational complexity analysis of DLGP algorithm.

Component	Per Iteration	Total (K Iterations)
**Computation**		
Local gradient computation	$O(nt_{\max}md)$	$O(Knt_{\max}md)$
Local Hessian computation	$O(nt_{\max}md^{2})$	$O(Knt_{\max}md^{2})$
Newton update	$O(nt_{\max}d^{3})$	$O(Knt_{\max}d^{3})$
Sensitivity calculation	O(n)	O(Kn)
Noise generation	O(nd)	O(Knd)
Global aggregation	O(nd)	O(Knd)
Dual variable update	O(nd)	O(Knd)
**Communication**		
Parameter transmission	O(nd)	O(Knd)
**Total Complexity**	$O\left(Knt_{\max}\left(md^{2}+d^{3}\right)+Knd\right)$	

**Table 3:** Default experimental parameters.

Parameter	Default Value
Iterations (*K*)	100
Edge nodes (*n*)	100
Penalty parameter (μ)	0.1
Privacy budget (ϵ)	0.9
Relaxation probability (δ)	0.01
Regularization parameter (λ)	0.01

**Table 4:** Empirical loss comparison with different numbers of nodes.

Algorithm	ϵ = 0.5				ϵ = 0.9			
	n ** = 50**	n ** = 80**	n ** = 100**	n ** = 200**	n ** = 50**	n ** = 80**	n ** = 100**	n ** = 200**
Centralized	0.0451	0.0451	0.0451	0.0451	0.0451	0.0451	0.0451	0.0451
ADMM	0.0455	0.0461	0.0464	0.0471	0.0455	0.0461	0.0464	0.0471
DLVP	0.4897	1.1639	1.1625	3.3431	0.1861	0.4415	0.4906	1.5274
DPSGD	0.3502	0.5257	0.5592	0.6685	0.3694	0.3971	0.4415	0.4906
DLGP	0.0840	0.2050	0.2075	0.6509	0.0572	0.0895	0.0943	0.2705
DP-PSAC-FL	0.3150	0.4832	0.5033	0.6679	0.3171	0.3176	0.3837	0.4564

**Table 5:** Accuracy comparison with different numbers of nodes.

Algorithm	ϵ = 0.5				ϵ = 0.9			
	n ** = 50**	n ** = 80**	n ** = 100**	n ** = 200**	n ** = 50**	n ** = 80**	n ** = 100**	n ** = 200**
Centralized	0.9915	0.9915	0.9915	0.9915	0.9915	0.9915	0.9915	0.9915
ADMM	0.9915	0.9911	0.9911	0.9911	0.9915	0.9911	0.9911	0.9911
DLVP	0.9782	0.9673	0.9561	0.9031	0.9906	0.9797	0.9685	0.9154
DPSGD	0.9029	08960	0.8862	0.8423	0.9153	0.9083	0.8985	0.8545
DLGP	0.9870	0.9791	0.9739	0.9498	0.9911	0.9821	0.9766	0.9510
DP-PSAC-FL	0.9372	0.9315	0.9209	0.8819	0.9461	0.9348	0.9177	0.8576

**Table 6:** Average computation time of algorithms.

Algorithm	Average Runtime (s)
ADMM	2.0768
DLVP	2.2812
DPSGD	0.2471
DLGP	2.1555
DP-PSAC-FL	0.3158

## Data Availability

The original data can be accessed from the UCI Machine Learning Repository at https://archive.ics.uci.edu/dataset/864 (accessed on 30 October 2025).

## References

[1] Yang Z., Xia W., Lu Z., Chen Y., Li X., Zhang Y. (2025). Hypernetwork-Based Physics-Driven Personalized Federated Learning for CT Imaging. IEEE Trans. Neural Netw. Learn. Syst..

[2] Wang C.C., Chien C.H. (2025). Machine Learning for Industrial Optimization and Predictive Control: A Patent-Based Perspective with a Focus on Taiwan’s High-Tech Manufacturing. Processes.

[3] Yuan F., Wang K., Ying J., Hou R., Zhao L., Li P., Zhu Y., Ji Z., Meng D. (2023). Architecting the Autocuckoo Filter to Defend Against Cross-Core Cache Attacks. IEEE Trans.-Comput.-Aided Des. Integr. Circuits Syst..

[4] Behmanesh M., Adibi P., Ehsani S.M.S., Chanussot J. (2024). Geometric Multimodal Deep Learning With Multiscaled Graph Wavelet Convolutional Network. IEEE Trans. Neural Netw. Learn. Syst..

[5] Lin Z., Lin H., Lin L., Chen S., Liu X. (2025). Robust cross-image adversarial watermark with JPEG resistance for defending against Deepfake models. Comput. Vis. Image Underst..

[6] Ruby R., Yang H., de Figueiredo F.A.P., Huynh-The T., Wu K. (2023). Energy-Efficient Multiprocessor-Based Computation and Communication Resource Allocation in Two-Tier Federated Learning Networks. IEEE Internet Things J..

[7] Ma Q., Xu Y., Xu H., Liu J., Huang L. (2024). FedUC: A Unified Clustering Approach for Hierarchical Federated Learning. IEEE Trans. Mob. Comput..

[8] Dwork C., Rothblum G.N., Vadhan S. Boosting and Differential Privacy. Proceedings of the 2010 IEEE 51st Annual Symposium on Foundations of Computer Science.

[9] Dwork C. The Promise of Differential Privacy: A Tutorial on Algorithmic Techniques. Proceedings of the 2011 IEEE 52nd Annual Symposium on Foundations of Computer Science.

[10] Ramakrishna R., Scaglione A., Wu T., Ravi N., Peisert S. (2023). Differential Privacy for Class-Based Data: A Practical Gaussian Mechanism. IEEE Trans. Inf. Forensics Secur..

[11] Liu F. (2019). Generalized Gaussian Mechanism for Differential Privacy. IEEE Trans. Knowl. Data Eng..

[12] Yuan J., Wnag Y., Ji Z. (2020). A differentially private square root unscented Kalman filter for protecting process parameters in ICPSs. ISA Trans..

[13] Zhang H., Li K., Huang T., Zhang X., Li W., Jin Z., Gao F., Gao M. (2023). Publishing locally private high-dimensional synthetic data efficiently. Inf. Sci..

[14] Sabah F., Chen Y., Yang Z., Azam M., Ahmad N., Sarwar R. (2024). Model optimization techniques in personalized federated learning: A survey. Expert Syst. Appl..

[15] Huang Z., Hu R., Guo Y., Chan-Tin E., Gong Y. (2020). DP-ADMM: ADMM-Based Distributed Learning With Differential Privacy. IEEE Trans. Inf. Forensics Secur..

[16] Liu Y., Geng J., Shang F., An W., Liu H., Zhu Q., Feng W. (2022). Laplacian Smoothing Stochastic ADMMs With Differential Privacy Guarantees. IEEE Trans. Inf. Forensics Secur..

[17] Zhao D., Zhang C., Cao X., Peng C., Sun B., Li K., Li Y. (2023). Differential Privacy Energy Management for Islanded Microgrids With Distributed Consensus-Based ADMM Algorithm. IEEE Trans. Control Syst. Technol..

[18] Zhang T., Zhu Q. (2017). Dynamic Differential Privacy for ADMM-Based Distributed Classification Learning. IEEE Trans. Inf. Forensics Secur..

[19] Wang X., Ishii H., Du L., Cheng P., Chen J. (2020). Privacy-Preserving Distributed Machine Learning via Local Randomization and ADMM Perturbation. IEEE Trans. Signal Process..

[20] Zhang F., Xue E., Guo R., Qu G., Zhao G., Zomaya A.Y. (2022). DS-ADMM++: A Novel Distributed Quantized ADMM to Speed up Differentially Private Matrix Factorization. IEEE Trans. Parallel Distrib. Syst..

[21] Kouhounestani M., Lee W. (2022). Datalog Static Analysis in Secrecy. IEEE Access.

[22] Shi Y., Nekouei E. (2024). Secure Adaptive Control of Linear Networked Systems Using Paillier Encryption. IEEE Trans. Circuits Syst. Regul. Pap..

[23] Cheng A., Wang P., Zhang X.S., Cheng J. Differentially Private Federated Learning with Local Regularization and Sparsification. Proceedings of the 2022 IEEE/CVF Conference on Computer Vision and Pattern Recognition (CVPR).

[24] Wang P., Zhang H. (2020). Distributed logistic regression with differential privacy. Sci. China Inf. Sci..

[25] Zhang C.J., Shan G.Y., Roh B.H. Communication-efficient federated multi-domain learning for network anomaly detection. Digit. Commun. Netw..

[26] Guo X., Chang T., Wang Y. (2023). Model-Driven Deep Learning ADMM Decoder for Irregular Binary LDPC Codes. IEEE Commun. Lett..

[27] Azimi-Abarghouyi S.M., Bastianello N., Johansson K.H., Fodor V. (2025). Hierarchical Federated ADMM. IEEE Netw. Lett..

[28] Singh A.P., Jain V., Chaudhari S., Kraemer F.A., Werner S., Garg V. Machine Learning-Based Occupancy Estimation Using Multivariate Sensor Nodes. Proceedings of the 2018 IEEE Globecom Workshops (GC Wkshps).

[29] Abadi M., Chu A., Goodfellow I., McMahan H.B., Mironov I., Talwar K., Zhang L. Deep Learning with Differential Privacy. Proceedings of the 2016 ACM SIGSAC Conference on Computer and Communications Security.

[30] Yuan J.Y., Chen Y. (2025). Differential Private Federated Learning with Per-Sample Adaptive Clipping and Layer-Wise Gradient Perturbation. Comput. Netw..

